# Methadone clinic staff perceptions of trauma-informed and patient-centered care: the role of individual staff characteristics

**DOI:** 10.1186/s13722-024-00501-6

**Published:** 2024-12-01

**Authors:** Beth E. Meyerson, Linnea B. Linde-Krieger, Gregory A. Carter, Allison J. Huff, Benjamin R. Brady, Richard A. Crosby, Jennifer De La Rosa, Allie Allison, Mohammad Barakat, Michael Pava, Mark Schaefer

**Affiliations:** 1 Harm Reduction Research Lab, Department of Family and Community Medicine, University of Arizona College of Medicine-Tucson, 655 N. Alvernon Way, Tucson, AZ 85711 USA; 2 Comprehensive Center for Pain and Addiction, University of Arizona Health Sciences, Tucson, AZ USA; 3 Department of Family and Community Medicine, University of Arizona College of Medicine-Tucson, Tucson, AZ USA; 4 Indiana University School of Nursing, Indiana University, Bloomington, IN USA; 5 School of Interdisciplinary Health Programs, College of Health and Human Services, Western Michigan University, Kalamazoo Michigan, USA; 6 University of Kentucky College of Public Health, Lexington Kentucky, USA; 7 Community Medical Services, Phoenix, AZ USA

**Keywords:** Opioid treatment program, Medication for opioid use disorder, Treatment practice change, Methadone, Trauma-informed care, Patient-centered care

## Abstract

**Background:**

U.S. policy intervention to increase methadone treatment accommodations during COVID did not result in national adoption of the new patient-centered treatment practices. Staff-level interventions may facilitate adoption of these treatment practices, but this will depend upon knowledge about staff level characteristics and beliefs. Currently, the role of clinic staff characteristics, beliefs about patient-centeredness, and perceptions about the need for treatment practice change is unknown. This study explored the relationship between opioid treatment program staff characteristics, work roles and staff beliefs to identify opportunities for future staff-level treatment practice change interventions.

**Methods:**

Staff of three Arizona opioid treatment programs were surveyed (n = 40) from April 11–22, 2023 using a hybrid online survey method. The 161 survey items required less than 30 min to complete. Pearson point biserial correlation coefficients assessed the covariation between staff beliefs, staff characteristics and staff work roles. Perception of the clinic as person-centered was a potential proxy indicator for staff awareness of discontinuity between the clinic’s person-centeredness and person-centered approaches to methadone treatment.

**Results:**

Among staff, 47.5% reported lived substance use disorder experience and 27.5% reported lived opioid use disorder experience. Most staff (70%) held at least 1 prior clinic role at the current clinic and 5% had had more than 4 prior roles. Rotation was observed with roles that did not require licensure or degrees. Staff with lived experience with substance use disorder or opioid use disorder treatment reported having more prior roles at the clinic than those without such experience. Abstinence-oriented views were significantly associated with reporting vicarious (work related) trauma symptoms. Those who rated the clinic as significantly more person-centered were staff with lived substance use disorder experience who also held abstinence-oriented views, staff with trauma exposure, and staff with lived opioid use disorder treatment experience who held harm reduction beliefs. In contrast, staff without substance use disorder experience who held harm reduction beliefs perceived the clinic as less person-centered.

**Conclusions:**

Staff beliefs, personal and work characteristics are likely factors in the recognition of need for clinic practice change. How these characteristics function in a clinic culture may also be influenced by clinic staffing patterns. A patient-to-provider pipeline with role cycling was observed and this staffing pattern may also influence shared beliefs of trauma-informed care or clinic person-centeredness. Vicarious trauma may also be an important factor. Larger studies should examine these relationships further to understand mechanisms associated with recognition of need for clinic practice change in order to inform staff-level interventions to increase opioid treatment program patient-centeredness.

## Background

Methadone and buprenorphine are effective treatments for opioid use disorder (OUD) and have also been found to reduce overdose and all-cause mortality [1, 2]. Both medications are safe and yet, in the U.S., are delivered in entirely different ways due to bifurcated federal regulatory regimes [3–5]. Methadone, if treating OUD and not pain, can only be delivered by certified opioid treatment programs (OTPs, ‘methadone clinics’) which operate with stringent federal regulatory oversight and varied state regulatory constraints [6]. This is in contrast with methadone as pain treatment and buprenorphine as OUD treatment; both of which can be prescribed in medical offices or clinics and dispensed by pharmacies [7]. These regulatory and treatment delivery differences are unique artifacts of the U.S. healthcare policy landscape and not found among international peers such as Australia, Switzerland, or the United Kingdom [8, 9].

U.S. methadone maintenance treatment (MMT) outcomes have been suboptimal, with patient treatment retention as low as 30% [10, 11]. It is increasingly recognized that OTP clinic practices may themselves contribute to these outcomes. OTPs are known not to be patient-centered with individuated care. Instead, they are known to have stigmatizing and inflexible, ‘one size fits all’ treatment protocols. Currently 2,147 U.S. OTPs serve an estimated 524,160 patients, with a median patient census of 260 and daily patient clinic average of over 100 for supervised ingestion of liquid methadone [12–14]. This daily crush of clinic patients appears to be the norm, as 50% of patients report never receiving more than a one- or two-day supply of medication (multiday dosing or ‘take homes’). This means  that they must come to the clinic several times a week, wait in line for (sometimes) hours to take their medication under the supervision  a dosing nurse at the clinic [15, 16]. The dosing language also distinguishes MMT from mainstream healthcare by referring to multiday medication not as a prescription but as “privileges.”[17, 18] Patients receiving multiday doses may be required to participate in “bottle counts”[19] whereby the clinic interrupts them at random to demand all doses be brought to the clinic within 1 h [20]. Some OTPs supervise urine drug screens directly or indirectly (with cameras), [21] despite general awareness that OUD patients have levels of sexual trauma histories and symptoms surpassing the general population [22, 23]. Notably, these practices are not evidence-based or patient-centered (individuated by patient unique circumstance and need); yet they appear to be uniformly required. Extant studies of these practices found they contribute to increased patient stigma, [24, 25] burden, [9, 26] and adverse MMT outcomes [18, 20].

It is not entirely clear why evidence-based, patient-centered practices are not fully embraced by OTPs system-wide. A multiyear national study of OTP practice changes between 1988 and 2000 found that while practices are slowly resembling the evidence-base, they were not fully implemented, especially for programs serving African American patients [27]. Federal and state regulations have been blamed for the way OTPs provide MMT [21]. During the COVID pandemic, federal policy allowed significantly enhanced MMT flexibility including multiday dosing of up to 14 days for unstable patients and up to 28 days if patients were deemed stable by their providers [28]. This was followed by planned patient-centered regulatory flexibilities made permanent in 2024 by the Final Rule, 42 CFR §8 [29]. Despite allowed flexibilities during COVID, studies found that while OTPs increased multiday dosing during COVID and particularly during state shut down periods, full alignment and sustaining of these treatment accommodations depended upon location [30–32]. Most OTPs expanded their use of telehealth to deliver MMT due to state regulatory payment parity, [33–35, 40] however an Arizona study found that MMT delivery emphasized OTP organizational priorities over patient safety, as > 50% of patients at risk for severe COVID outcomes were still required to attend daily clinic-supervised dosing [42].

Collectively, the understudied environment of OTPs, the reported uniformity of OTP practices that are not patient-centered, and the fact that federal policy did not appreciably change these practices necessitate the examination of clinic and staff-level factors such as OTP practice culture and staff characteristics. If external policy did not facilitate change toward patient-centered practices, perhaps at the clinic level (culture and staff) there may be important factors for change. For example, a 2022 study among a sample of New York “substance use service” programs highlighted the potential difference in the setting’s culture, as OTPs were less likely than other types of substance use service programs to support multiday dosing [36].

Recognizing the need for change has been noted as an important staff characteristic to facilitate adoption of new practices [37]. A few studies point provide foundational evidence.  A 2023 study found that individual characteristics of substance use disorder treatment (SUDtx) program staff weakened the ability to predict needed change and respond to change [38]. Other studies noted the role of SUDtx staff stigma and abstinence orientations on staff practice behaviors and beliefs about them [39–42].

Finally, characteristics influencing practice change may include staff trauma histories and symptoms. Though this is not established for the OTP environment, other high stress treatment fields provide some indication. Domestic violence counselors with trauma histories were more likely to experience vicarious (work related) trauma [43, 44] which both increased staff turnover and decreased productivity [45]. Notably, vicarious trauma has been found to alter provider world view and beliefs about themselves and their patients [46]. This suggests that vicarious trauma is likely critical to recognizing the need for change and being open to it, as suggested by one study finding that vicarious trauma among SUDtx staff was associated with lower patient empathy, patient-centeredness and a resistance to practice change [47]. In the case of recent federal policy to facilitate adoption of treatment accommodations among OTPs, the recognition of need for practice change may be related to the perceptions of person-centeredness in the OTP setting itself.

Several studies note higher trauma rates among people on MMT [48–50]. This fact, as well as requirements of high patient-volume OTPs with stringent rules, may contribute to vicarious trauma among OTP staff. A 2023 survey among staff of three Arizona OTPs offered preliminary confirmation of vicarious trauma among OTP staff, finding moderate levels of vicarious trauma reported by 60.0% of staff and high levels reported by 13%. Further, most staff (70.0%) reported at least four adverse childhood experiences, and 63.0% had clinically significant post-traumatic stress symptoms [51]. The study reported here further explores the association between OTP individual staff characteristics including trauma and perceptions of clinic person-centeredness as a proxy for recognizing the need to change toward greater patient-centeredness. The rationale for conceptualizing recognition of need for change through a proxy indicator of perceptions of clinic person-centeredness is based on observations that current clinic practices are not individuated but ‘one size fits all,’ and that beliefs about more individuated practices inherent in person-centeredness would suggest an important philosophical discordance between staff belief and clinic climate. Use of other organizational need for change measures would not be as precise in terms of indicating need for clinic practice change related to person-centeredness or individuated care.

## Methods

Study aims were to examine the association of OTP staff characteristics of SUD experience, trauma history and symptoms, and demographics; work characteristics; and beliefs about harm reduction, trauma informed care and patient-centeredness of the clinic.

Staff of three Arizona OTPs were surveyed from April 11-22, 2023. One clinic was located in a large rural town in southern Arizona (45,000 population) and served several smaller, surrounding areas. This clinic’s patient population was 200, and 10 people staffed the clinic. The other two clinics were located in urban areas (over 500,000 population) and served 900 to 1, 000 patients respectively; each with 20 staff members. An anonymous survey was fielded to all staff of the three OTPs using a hybrid (paper to online) method employed previously with pharmacists, [52, 53] nurses, [54] and medication providers for opioid use disorder [40, 55].

A survey recruitment flyer for voluntary and anonymous survey participation was posted in staff-only areas of the participating clinics. The flyer provided information about the survey, the incentive for completion ($40) and a QR code and URL to link interested participants to a landing page containing study information, informed consent and, if consented, the survey itself. The survey contained 161 items and required up to 30 min to complete. Study oversight was provided by the University of Arizona Institutional Review Board.

### Measures

The survey instrument measured demographics, staff work characteristics, personal characteristics and beliefs. Work characteristics included time working at the specific OTP, time working in SUDtx, current self-reported role at the clinic, and other roles previously held at the same clinic. Personal characteristics included demographics, personal SUD experience, personal OUD experience, personal experience with a list of possible OUD treatments (methadone, buprenorphine, naltrexone, or other), and trauma history and symptoms.

Beliefs about trauma-informed care (what constitutes it) were measured by the validated 10-item short form of the Attitudes Related to Trauma-Informed Care scale (ARTIC-10) [56]. Average ARTIC-10 scores were used in these analyses (α = 0.80). Opioid related stigma was measured by one item from the Brief Opioid Stigma Scale: *I believe that a person who is addicted to opioids cannot be trusted* [57]. Trauma was measured in three ways: (1) trauma histories by the Life Events Checklist (LEC-5)[58] and the Adverse Childhood Experiences (ACES) scale [59]; (2) trauma symptoms by the PTSD Checklist for the DSM-5 (PCL-5)[60] and the Trauma Symptoms Checklist (TSC-40) [61]; and (3) vicarious trauma by the Vicarious Trauma Scale [62]. Finally, perceptions of the degree to which the OTP was patient-centered were measured on the 14-item Person-Centered Climate Scale-Staff Version (PCQ-S) [63–65].

The survey also contained three open-ended questions: (a)* please share what “patient-empowered methadone treatment” means to you*; (b) *please share what “trauma-informed methadone treatment” means to you*; and (c) *if you could wave a magic wand, how would you improve methadone treatment*? These questions were coded to further understand staff conceptualizations of what constituted trauma-informed MMT, patient-centered MMT and to characterize the expressions of abstinence-oriented and harm reductive viewpoints. For clarity, there were two measures related to patient or person-centeredness: (1) staff perceptions of the person-centeredness of the clinic environment measured by the PCQ-S scale and (2) staff perceptions of what constitutes patient-centered methadone treatment (as a concept) which was qualitatively measured.

### Data analysis

Descriptive statistics characterized OTP staff demographics, clinic roles, and lived experiences of SUD and OUD treatment. Chi-square tests measured associations among dichotomous study variables including staff lived experience of any SUD (yes/no), staff lived experience of OUD treatment (coded yes for OUD treatment selected/no), and clinic role (counselor vs. other) with treatment views (i.e., harm reduction views, trauma-informed views, patient-centered views, and abstinence-oriented views). Dichotomous variables of these views resulted from the quantitation of qualitatively coded text from themes emerging from the three open-ended questions. ‘Yes’ would indicate views were present. Pearson point biserial correlation coefficients assessed the magnitude and direction of bivariate associations between staff treatment views and continuous study variables in the total sample, separately for staff with lived SUD experience and for staff with OUD treatment experience.

Qualitative data were coded using a priori categories of ‘patient-centered’ and ‘trauma-informed’ to organize emerging themes. This was followed by an open coding approach to allow the occurrence of additional themes from the data which permitted the creation of an array of themes and their relationship through the identification of philosophical connections in the rich descriptions of concepts by respondents. For rigor, a sample of 21 survey responses were independently coded by three investigators followed by a coding conference resulting in the final scheme used to code all qualitative data. Finally, respondent statements were quantitated into “harm reduction” (yes/no) and “abstinence-oriented” (yes/no) for analysis across cases and comparison with other study measures. These categories were not mutually exclusive, as one participant responses could be coded as harm reduction oriented and another from the same participant as abstinence oriented. The basis for coding a response as harm reduction-oriented was based on harm reduction principles articulated by the National Harm Reduction Coalition [66]. Statements were coded as abstinence-oriented if expressing a goal of methadone cessation, or full cessation of any substance as a goal. Quantitative analyses were conducted in SPSS v.28 and qualitative analyses were conducted in QSR NVIVO v.14.23. Comparative results significant at the p < 0.05 level were reported unless otherwise noted.

## Results

Forty staff from the three OTP clinics participated in the survey for a response rate of 80%. As shown in Table 1, participants were diverse with regard to age, time working in SUD treatment, race, and ethnicity. Nearly half of respondents (47.5%) reported having personal lived experience with SUD and 27.5% of respondents reported a history of OUD treatment, including 12.5% reporting methadone treatment and 10% reporting buprenorphine treatment.

**Table 1:** Opioid treatment staff characteristics in three Arizona clinics, 2023 (N = 40)

	Mean (SD)	Range
Age (years)	37.9 (10.5)	22—59
Years working in SUD treatment	3.6 (3.1)	0—15
Years working at current clinic	1.5 (1.5)	0—6
Number of prior roles at current clinic	1.1 (1.0)	0—4

	%	N
Race/Ethnicity*		
White	57.5	23
Black	12.5	5
Other race	25.0	10
Latinx/Hispanic ethnicity	37.5	15
Prefer not to say	5.0	2
Clinic Role		
Nurse	15.0	6
Leadership (clinic manager, site supervisor, clinical coordinator)	10.0	4
Counselor	40.0	16
Peer support (peer support specialist, patient navigator)	15.0	6
Case manager	5.0	2
Front desk staff	12.5	5
Prefer not to say	2.5	1
Lived SUD experience		
Personal experience with any SUD	47.5	19
Personal experience with OUD treatment	27.5	11
Personal experience with methadone treatment	12.5	5
Personal experience with buprenorphine treatment	10	4
Prefer not to say	10	4

^*^No participants identified as Asian, Native Hawaiian or Pacific Islander, or American Indian/Native Alaskan

### Work characteristics

As shown in Table 1, staff in counseling roles comprised 40% of the sample. Medical providers responding to the survey were all nurses (i.e., RN, LPN, NP), and they comprised 15% of the sample. Finally, 15% of staff were in peer support or patient navigator roles. Administrative roles included front desk staff (12.5%) and clinic leadership (10.0%). Staff with lived SUD experience were more likely to be in a counselor role than staff without SUD experience (47.4% vs. 29.4%). Only one person in a medical role reported SUD lived experience.

Roles transition within the OTP was observed. The majority of respondents (70%) reported at least one prior role at their treatment clinic. In terms of number of previously held roles, 45% of staff reported having held one prior role at the same clinic, 17.5% had two, 2.5% had 3, and 5% had held 4 prior roles for a mean number of prior roles at the same clinic of 1.08. Transition appeared to involve roles that did not require particular degrees or certifications, including the counseling role. Certifications (degrees and licensure) are not required for counselors in OTPs by federal regulation [7] or, to our knowledge, state regulation, [6] though particular OTP companies may have specific requirements. Fifteen percent of all respondents reported that they had previously worked as case managers, 25% reported that they had previously worked as front desk staff, and 10% reported that they had previously worked as peer support specialists at the same clinic. Former case managers tended to move into counseling roles (i.e., 25% of counselors previously held a position of case manager at the same clinic), and former front desk staff tended to move into roles of navigator, case manager, or counselor. Among former peer support specialists, two moved into a counseling role, one moved into a clinic leadership role, and one moved into a medical provider role. Notably, staff members who reported lived experience with SUD held significantly more prior roles (m = 1.47) in the same clinic than staff without lived SUD experience (m = 0.71, t(34) = 2.36, p = 0.02). This finding also held for staff members with lived experience of OUD treatment (m = 1.82) compared with those without lived OUD treatment experience (m = 0.82, t(37) = 3.02, p = 0.005), suggesting that staff with lived SUD experience, and OUD treatment experience in particular, occupied more work roles over time at the same clinic than staff without lived experience of SUD or OUD treatment.

### Trauma outcomes

A vast majority of staff (90%) indicated personal experience of at least one significant traumatic event during their lifetimes, and 57.5% had personally experienced four or more traumatic events. Experiencing 4 or more traumatic events means that a person was at high risk for toxic stress [67]. The average number of personally experienced lifetime traumatic events was 5.13 (SD = 3.69, range: 0–14). The average number of witnessed traumatic events was 3.58 (SD = 4.38, range: 0–14). Vicarious trauma exposure was similarly pronounced, with 82.5% of respondents reporting that their job requires exposure to distressed or traumatized clients, and 65% reporting that their job involves exposure to distressing materials and experiences. Notably, 62.5% of respondents PTSS measures in the clinical range (i.e., provisional PTSD diagnosis), 12.5% had high levels of vicarious trauma, and 60% had moderate levels of vicarious trauma.

### Staff beliefs

Most staff (77.5%) disagreed with the statement *I believe that a person who is addicted to opioids cannot be trusted*. Qualitative harm reduction-oriented views were also reported by 50% of staff through statements such as:

We are meeting clients where they are. Allowing clients to take charge of their treatment, setting goals that are important to them. Find ways to incorporate more harm reduction approaches and therapy. (Case 31)

These views contrasted with the 15% of staff who articulated abstinence-oriented views. These views tended to express inflexibility with treatment requirements, the goal of complete abstinence from other drugs during MMT, as well as eventual abstinence from methadone altogether. Exemplar statements were such as:

(what) patient empowered methadone treatment means to me is to educate a person to never use methadone in large amounts or for longer than prescribed. (Case 5)

….be a bit more strict with clients treatment. I understand we deal with clients on a case by case basis but there must be a place in our program where we can say across the board that this borders on enabling not helping this client. There is no concrete line for that across the board and if there is no standard, can there really be order? (Case 8)

**Fig. 1:**
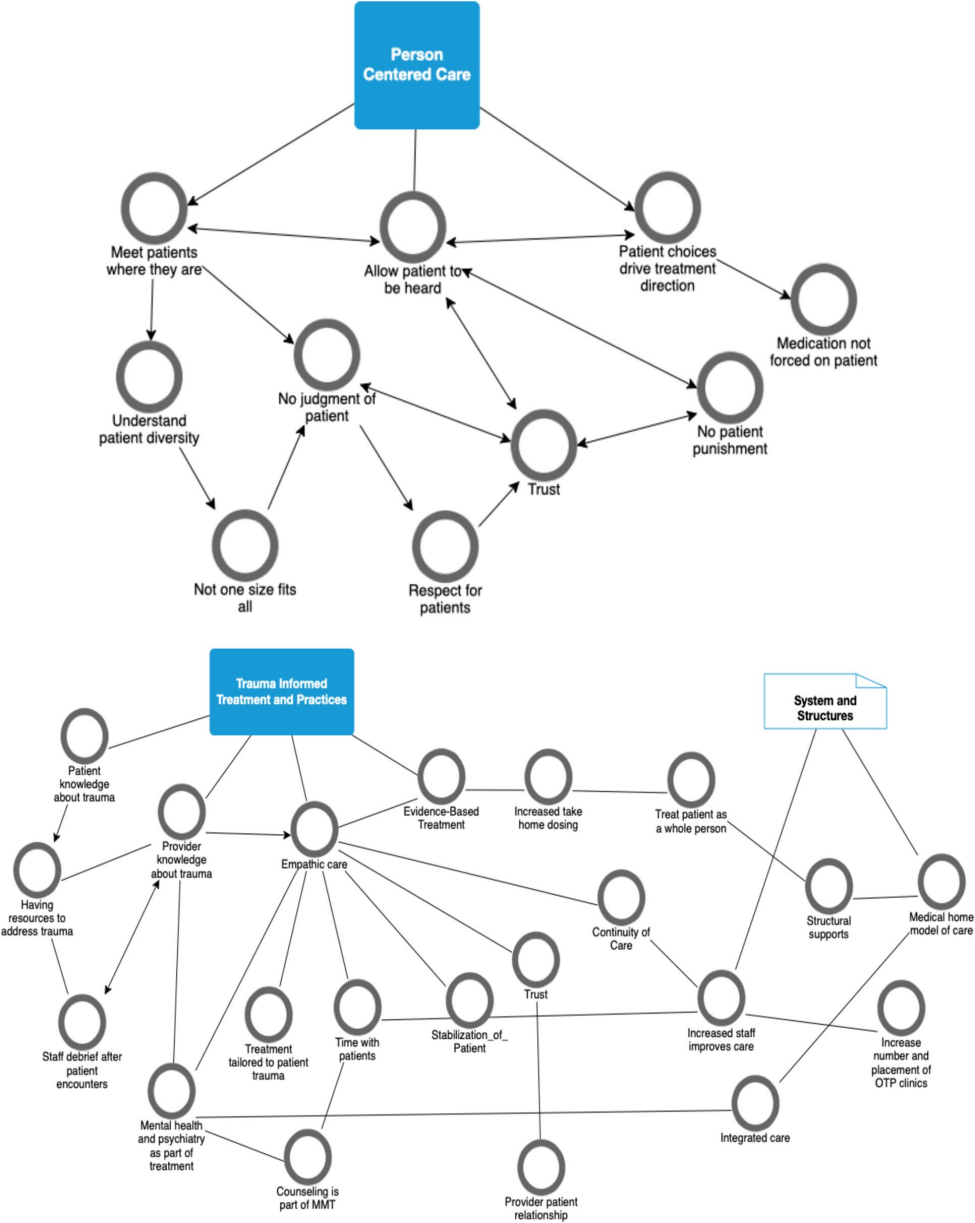
Emerging themes of person-centered MMT and trauma-informed MMT among OTP staff of three Arizona clinics, 2023 (N = 40)

Figure 1 depicts the themes emerging from respondents’ written comments about what constituted patient-empowered MMT and what constituted trauma-informed MMT. Arrows represent connections assigned by the research team based on analysis of written response and connections made among concepts by participants. A vast majority of staff (70%) expressed patient-centered views. Major themes included the patient being in control, approaches that were harm reductive, flexible treatment options and approaches, trust between patient and provider, and respect for the patient.

Over half of staff (55%) held trauma-informed views. Major themes focused on two areas: practice and structures. The identified structures included the requirement that systems be in place to support trauma-informed care. Practice themes were evidence-based care, empathic care, and knowledge/learning for patients and providers about what constituted trauma-informed care. Qualitatively observed attitudes were reflected in responses to the validated ARTIC scale measuring favorability or unfavourability related to trauma informed care. The ARTIC mean score was 5.4 out of 7 indicating a very favorable view of trauma informed care.

Staff also reported beliefs about the person-centered climate or environment of the OTP. Just over half (55%) agreed that the OTP climate was person centered overall, and the average PCQ-S item score was 4.3 out of 6 ( “agree”); but there was wide variation in staff perceptions of clinic environment; with average PCQ-S item scores ranging from 2 (“strongly disagree”) to 6 (“very strongly agree”). The average PCQ-S total score was 51.6 (SD:10.1, r:25–72). Staff with lived SUD experience rated the OTP clinic environment as significantly more person centered (*m* = 4.68) than staff without lived SUD experience (*m* = 4.02), t(33) = 2.7, *p* = 0.01).

### Connections between and among characteristics and beliefs

There were no statistically significant associations between having SUD experience and qualitative views about treatment. Staff with lived SUD experience were not more likely than staff without lived SUD experience to express views about harm reduction (52.6% vs. 47.1%, *X*^2^(1) = 0.11, *p* = 0.74), trauma-informed care (57.9% vs. 52.9%, *X*^2^(1) = 0.09, *p* = 0.77), patient-centered care (63.2% vs. 76.5%, *X*^2^(1) = 0.75, *p* = 0.39), or abstinence beliefs (10.5% vs. 11.8%, *X*^2^(1) = 0.01, *p* = 0.91). Similar outcomes were observed for staff with lived OUD treatment experience. Specifically, these staff were not more likely to hold views about harm reduction (63.6% vs. 42.9%, *X*^2^(1) = 1.37, *p* = 0.24), trauma-informed care (54.5% vs. 57.1%, *X*^2^(1) = 0.02, *p* = 0.88), patient-centered care (63.6% vs. 75%, *X*^2^(1) = 0.50, *p* = 0.48), or abstinence beliefs (9% vs. 14.3%, *X*^2^(1) = 0.19, *p* = 0.66) compared with staff without OUD treatment experience. Similarly, staff in counseling roles and staff in other roles (non-counselor) did not differ in their expression of views about trauma-informed care (56.3% vs. 54.2%, *X*^2^(1) = 0.02, *p* = 0.90), patient-centered care (62.5% vs. 75%, *X*^2^(1) = 0.71, *p* = 0.40), or abstinence beliefs (12.5% vs. 16.7%, *X*^2^(1) = 0.13, *p* = 0.72). However, there was a marginally significant relationship found between staff in counseling roles and having harm reduction views as compared with staff not in counseling roles (68.8% vs. 37.5%, *X*^2^(1) = 3.75, *p* = 0.05).

Bivariate correlations in the total sample revealed that trauma exposure was positively associated with having trauma symptoms, rating the clinic climate as person-centered, and having trauma-informed beliefs. As shown in Table 2, there was a positive association of trauma-informed beliefs on the ARTIC and ratings of OTP person centeredness on the PCQ-S. Age was positively associated with trauma exposure and years working in SUD treatment, and negatively associated with vicarious trauma symptoms, indicating that older staff members had experienced more traumatic events but were less affected by vicarious trauma in the workplace.

**Table 2:** Bivariate correlations between staff characteristics, work characteristics and beliefs about the OTP, trauma-informed care and patient centered care, Arizona 2023 (N = 40)

	*M* (*SD*)	Age	Years working in SUDtx	Years at current clinic	Lifetime trauma exposures	Posttraumatic stress symptoms	Vicarious trauma symptoms	Person centered OTP climate	Stigma	Trauma informed Beliefs
1. Age (years)	37.92 (10.49)	–								
2. Years working in SUD treatment (SUDtx)	3.60 (3.06)	0.484**	–							
3. Years at current clinic	1.53 (1.48)	0.224	0.146	–						
4. Lifetime trauma exposures	8.70 (7.11)	0.331*	0.181	− 0.024	–					
5. Posttraumatic stress symptoms	40.83 (16.41)	− 0.047	0.064	− 0.007	0.596**	–				
6. Vicarious trauma symptoms	21.35 (8.20)	− 0.370*	− 0.235	0.302^**+**^	-0.014	0.236	–			
7. Person centered OTP climate	51.59 (10.09)	0.072	− 0.200	− 0.171	0.485**	0.238	− 0.236	–		
8. Stigma	1.08 (0.27)	0.251	0.294	− 0.047	0.171	0.017	0.018	0.030	–	
9. Trauma-informed beliefs (ARTIC)	5.35 (1.07)	0.188	− 0.096	0.120	0.348*	0.161	0.135	0.353*	− 0.079	–

SUD = substance use disorder. OUD = opioid use disorder. OTP = opioid treatment program

^+^p < 0.1

^*^p < 0.05

^**^p < 0.01

Point biserial correlations compared the qualitatively derived categories of harm reduction views, abstinence-oriented views, patient-centered views and trauma informed views with work characteristics (time working in an SUD clinic and time at current clinic), trauma history and symptoms (lifetime trauma exposure, posttraumatic stress symptoms and vicarious trauma), and staff beliefs measured by validated instruments (person-centeredness of clinic climate, stigma and trauma-informed beliefs (ARTIC)). As shown in Table 3 we observed a significant positive association between qualitatively coded patient-centered views and vicarious trauma symptoms. In contrast, abstinence-oriented views were significantly and negatively associated with vicarious trauma symptoms. Harm reduction views were positively related to lifetime trauma exposure and negatively related to vicarious trauma symptoms, though these associations did not reach statistical significance.

**Table 3:** Significant relationships between staff characteristics, work characteristics and beliefs about the OTP and trauma-informed care and patient centered care by staff lived SUD experience, Arizona 2023 (N = 40)

			Work Characteristics		Staff Trauma Exposure and Symptoms			Staff Beliefs		
		Age	Time working in SUD treatment	Time at current clinic	Lifetime trauma exposure	Posttraumatic stress symptoms	Vicarious trauma symptoms	Person centered OTP climate	Stigma	Trauma-informed beliefs (ARTIC)
Harm reduction views	Lived SUD	0.613**	0.383	0.005	0.388	0.100	− 0.552*	0.428^**+**^	0.297	0.421^**+**^
	Lived OUD Tx	0.857**	0.113	− 0.237	0.569*	0.075	− 0.513^**+**^	0.690*	0.439	0.477
Trauma-informed views	Lived SUD	− 0.047	0.074	0.182	− 0.220	− 0.181	0.280	− 0.101	− 0.077	0.647**
	Lived OUD Tx	− 0.124	− 0.230	0.049	− 0.310	− 0.229	0.507	− 0.267	0.064	0.725*
Patient-centered views	Full sample	− 0.242	− 0.141	− 0.010	0.050	0.239	0.520**	0.112	0.030	0.162
	Lived SUD	− 0.307	0.143	− 0.027	0.182	0.288	0.507*	0.087	0.202	0.160
	No Lived OUD Tx	− 0.229	0.025	− 0.016	0.046	0.311	0.533**	− 0.034	− 0.162	0.077
Abstinence-oriented views	Full sample	0.111	0.169	− 0.027	− 0.042	− 0.211	− 0.390*	0.075	0.045	− 0.218
	Lived SUD	0.297	0.081	− 0.060	0.115	− 0.035	− 0.528*	0.541*	− 0.166	0.004
	Lived OUD Tx	0.358	0.356	− 0.215	0.620*	0.217	− 0.208	0.557^**+**^	− 0.184	− 0.051

Staff experience was categorized as Full sample; Lived SUD, no lived SUD, lived OUD Tx, no lived OUD Tx. Associations found to be significant are reported here. The full table can be found in supplemental materials

SUD = substance use disorder. OUD = opioid use disorder. OTP = opioid treatment program

+ p < 0.1

^*^p < 0.05

^**^p < 0.01

Staff with SUD experience who held harm reduction views were older, had lower vicarious trauma symptoms, endorsed marginally higher trauma-informed beliefs on the ARTIC, and perceived the OTP as being marginally more person centered. In contrast, staff without SUD experience who held harm reduction views rated the OTP clinic as marginally less person centered (not reported in table). A similar pattern of results was observed with staff who reported OUD-specific treatment experience. Staff with OUD treatment experience who held harm reduction views were older, had greater lifetime trauma exposure, marginally lower vicarious trauma symptoms, and rated the OTP clinic as more person centered.

The addition of SUD experience appeared to factor into beliefs. Staff with lived SUD experience and staff with OUD-specific treatment experience who expressed trauma-informed views (qualitatively) were more likely to have trauma-informed beliefs on the ARTIC, but this association was not significant for staff without lived SUD experience. Staff with lived SUD experience who held abstinence-oriented views had lower vicarious trauma symptoms and rated the OTP climate as more person centered. Among staff with OUD-specific treatment experience, abstinence-oriented views were marginally associated with perceptions of the OTP climate as more person centered and significantly associated with trauma exposure. These associations were not significant for staff without lived SUD or OUD-specific treatment experience.

## Discussion

To our knowledge, this study is the first to explore the OTP staff characteristics (personal and work) and their association with beliefs about methadone treatment and the clinic person-centeredness. This study demonstrated the feasibility of survey research among OTP staff using a fairly lengthy instrument containing several scales measuring trauma histories, symptoms and vicarious trauma. The high response rate (80%) is likely an indicator of interest, though probably not what would be obtained in larger samples.

As noted elsewhere, [64] staff trauma histories and symptoms were likely related to reported SUD histories among staff. SUD experience may also be an important factor in two ways. On the one hand, SUD experience may be an important factor in the quality of care. For example, it may be that staff with SUD experience understand the patient experience in ways others might not. A nonsystematic review of studies published before 2010 of North American SUD providers identified that staff with their own histories of SUD may feel more closely aligned with their patients’ experiences [51]. Conversely, and by way of caution, closer personal alignment with patient experience could increase staff vulnerability to negative trauma-related outcomes (such as vicarious trauma symptoms). Staff experience with treatment, especially if counter to what is now considered evidence-based and patient-centered, could affect their beliefs about the treatment they now provide because they may believe that what they received in the past was standard of care. This would mean that they may not recognize their OTP as not being patient-centered. This, however, needs to be more fully explored. Further, when paired with unmanaged vicarious trauma symptoms, staff may seek psychological protections through a mantle of OTP practices that separate them further from patient trauma and need. This is relevant because this study documented that in this type of SUD clinic (the OTP setting vs. other SUD settings) there may be a higher percentage of staff with SUD histories. Extant studies among SUD treatment staff found that 1 in 5 have SUD and treatment histories [68, 69].

The combination of personal beliefs and experience were themselves related in interesting ways. Personal OUD treatment and SUD experience may moderate other beliefs. For example staff who rated the clinic as significantly more person-centered had lived SUD  experience and held abstinence-oriented views. Also, staff with lived OUD treatment experience who held harm reduction beliefs rated the clinic as more person-centered than those without the combination of these experiences and beliefs. Reasons for these initial observations are not entirely clear. While we did not measure the actual person-centeredness of the OTPs in this study, this finding raises the question of whether the combination of SUD and OUD treatment experience and harm reductive or abstinence-oriented beliefs somehow interrupts the recognition of need for greater person-centeredness in the clinic environment.

Understanding the observed relationships further will require improved measures such as the Abstinence Orientation Scale, a 14-point scale validated among OTP staff [70]. Given the potential role of staff lived SUD and OUD treatment experience in influencing their patient-centered and harm reduction views, it might also be prudent to incorporate a rating of received treatment for its abstinence orientation(s), and the degree to which it was trauma-informed or patient-centered. Identifying how to precisely measure that would greatly increase our knowledge about whether it is lived SUD experience and OUD treatment experience that matters or *lived bad OUD treatment experience* that matters (e.g. not trauma-informed or patient-centered).

A novel finding of this paper is the preliminary identification of a *sustained patient to provider pipeline* in methadone treatment which may (if shown in larger studies) constitute the majority of OTP staffing. While it is known that SUDtx fields increasingly use peers in supportive staff roles who were themselves prior patients, [71] we identified that a range of roles, including counseling, were part of this pipeline, and that staff with SUD experience tended to occupy more prior clinic roles than their counterparts. Again, this in and of itself may be a great strength, but it must be examined given the findings that SUD experience (perhaps in a larger sample it is SUD treatment experience) mediated perception of the clinic’s patient-centeredness (lack thereof), abstinence orientations, and the need for change to adopt more patient-centered views.

We also identified a work role cycling phenomenon. Work role rotation or cycling may also have important implications for OTP culture and change. Organizational cultures are established by the people within them and their socialization to the setting and one another [72]. If staff overall rotate through an organization *and remain there,* practice change may be impacted (for good or ill). Federal and state policy have some influence over the degree to which OTPs becoms more patient-centered and trauma-informed using evidence-based treatment practices, but it is likely that the inner setting of the OTP itself and the individual characteristics are also important factors influencing the implementation of established and evidence-based practices that can eliminate the current MMT suboptimal patient outcomes [73].

Study observations are preliminary and future studies should examine these relationships over time and in larger national samples across different types of OTP settings. With larger samples across many OTPs, the question may be whether lived MMT treatment or abstinence oriented OUD treatment is the deleterious factor that might, along with vicarious trauma and unaddressed trauma be most impactful when thinking about factors that influence recognition of the need for practice change and the readiness for it.

### Limitations

Observations from this study are preliminary and need to be understood over time and in a larger sample of staff in different OTP settings. A second limitation involved measurement. This study used only 1 item from a validated stigma scale as well as qualitative measures to gain deeper understanding of complex thinking about trauma-informed and patient-centered care. While qualitative measures were helpful to this particular study, larger studies should continue to use validated measures of patient-centeredness and trauma-informed beliefs. Third, the majority of the sample included people in counseling roles. This may have skewed findings, and efforts should be made first to assess the staff roles distribution in sample clinics and then stratify staff sampling by role  for more representative sampling. That said, as found in a prior study of Arizona MOUD providers, clinician response rates are low, [68] and there are no studies (to our knowledge) characterizing the roles distribution in U.S. OTP clinics. These issues notwithstanding, apparently all roles in the clinic (from front desk through providers) had the authority to place holds or ‘flags’ on methadone dosing [74]. This means that any staff can have control over a patient’s ability to manage their withdrawal symptoms through enforcement of OTP clinic rules that may or may not be evidence-based. An additional limitation is the use of the perception of clinic person-centeredness as a proxy indicator for clinic need for practice change. Perhaps a more reasonable conceptualization is the use of this measure as a proxy for need to change clinic culture; however, that would presume that staff want the clinic to be more person-centered and that the clinic should change to achieve that. Future cognitive interviews should be planned to help select the indicator for staff belief that the clinic needs to change and that practices in the clinic need to become more patient-centered. Finally, we did not investigate the professional training of staff beyond that of nursing training. Future studies will need to fully assess the myriad options for formal professional training (degrees and certificates) that are relevant to the delivery of MMT. This is significant, as it has been identified that ‘counseling’ in OTPs is rarely delivered by people who are trained clinically as counselors [21].

## Data Availability

The survey instrument is available upon request to the corresponding author and will involve the agreement that this published manuscript is cited when used in whole or in part. The anonymous survey data will not be available for request in order to protect human subjects. This is due to the small sample size and the ability to potentially de-identify the data based on role. This is particularly the case for the rural participants.

## Abbreviations

MMT: Methadone maintenance treatment or therapy
MOUD: Medication for opioid use disorder
SUD: Substance use disorder
OTP: Opioid Treatment Program, also known as methadone clinic
OUD: Opioid use disorder

## References

[1] Wakeman SE, Larochelle MR, Ameli O, Chaisson C, McPheeters JT, Crown WH, Azocar F, Sanghavi DM. Comparative effectiveness of different treatment pathways for opioid use disorder. JAMA Netw Open. 2020;3(2): e1920622. 32022884 10.1001/jamanetworkopen.2019.20622 PMC11143463

[2] Amato L, Davoli M, Perucci CA, Ferri M, Faggiano F, Mattick RP. An overview of systematic reviews of the effectiveness of opiate maintenance therapies: available evidence to inform clinical practice and research. J Subst Abuse Treat. 2005;28(4):321–9. 15925266 10.1016/j.jsat.2005.02.007

[3] Institute of Medicine Committee on Federal Regulation of Methadone Treatment. Federal Regulation of Methadone Treatment. In: Rettig RA, Yarmolinsky A, Eds. Federal Regulation of Methadone Treatment. National Academies Press; 1995:120–150. 25121195

[4] Harris J, McElrath K. Methadone as social control: institutionalized stigma and the prospect of recovery. Qual Health Res. 2012;22(6):810–24. 10.1177/1049732311432718. 22232295 10.1177/1049732311432718

[5] Drug Addiction Treatment Act, 1101, §3501. 2000. https://www.govinfo.gov/content/pkg/PLAW-106publ310/pdf/PLAW-106publ310.pdf. Accessed 24 Mar 2020.

[6] Pew Charitable Trusts. Overview of opioid treatment program regulations by state: restrictive rules put evidence-based medication treatment out of reach for many. September 2022. See https://www.pewtrusts.org/en/research-and-analysis/data-visualizations/2022/state-opioid-treatment-program-regulations-put-evidence-based-care-out-of-reach-for-many and https://www.pewtrusts.org/en/research-and-analysis/issue-briefs/2022/09/overview-of-opioid-treatment-program-regulations-by-state

[7] U.S. Substance abuse and mental health services administration. Federal guidelines for opioid treatment programs. Substance Abuse and Mental Health Services Administration; 2015.

[8] Henry-Edwards S, Gowing L, White J, Ali R, Bell J, Brough R, Lintzeris N, Ritter A, Quigley A. Clinical guidelines and procedures for the use of methadone in the maintenance and treatment of opioid dependence. Publications Production Unit, Australian Government Department of Health and Ageing, 2003; Publication approval number: 3263 (JN 7616).

[9] Calcaterra SL, Chadi BPA, et al. Methadone matters: What the United States can learn from the global effort to treat opioid addiction. J Gen Intern Med. 2019;34(6):1039–42. 30729416 10.1007/s11606-018-4801-3 PMC6544670

[10] Klimas J, Hamilton M-A, Gorfinkel L, Adam A, Cullen W, Wood E. Retention in opioid agonist treatment: a rapid review and meta-analysis comparing observational studies and randomized controlled trials. Syst Rev. 2021;10:216. 10.1186/s13643-021-01764-9. 34362464 10.1186/s13643-021-01764-9 PMC8348786

[11] Moradinazar M, Farnia V, Alikhani M, Karyani AK, Rezaei S, Rezaeian S, Matin BK, Najafi F. Factors related to relapse in patients with substance-related disorders under methadone maintenance therapy: decision tree analysis. Oman Med J. 2020;35(1): e89. 10.5001/omj.2020.07. 31993227 10.5001/omj.2020.07 PMC6976887

[12] Dunn KE, Brooner RK, Stoller KB. Technology-assisted methadone take-home dosing for dispensing methadone to persons with opioid use disorder during the COVID-19 pandemic. J Sub Abuse Treat. 2021;121: 108197. 10.1016/j.jsat.2020.108197. 10.1016/j.jsat.2020.108197 PMC7834258 33357606

[13] U.S. Substance Abuse and Mental Health Services Administration (SAMHSA). Opioid Treatment Program Directory. https://dpt2.samhsa.gov/treatment/directory.aspx Accessed 23 April 2024.

[14] U.S. Substance Abuse and Mental Health Services Administration, National Survey of Substance Abuse Treatment Services (N-SSATS): 2020. Data on Substance Abuse Treatment Facilities. Rockville, MD: Substance Abuse and Mental Health Services Administration, 2021

[15] Figgatt MC, Salazar Z, Day E, Vincent L, Dasgupta N. Take-home dosing experiences among persons receiving methadone maintenance treatment during COVID-19. J Subst Abust Treat. 2021;123:1–4. 10.1016/j.jsat.2021.108276 PMC8060693 33612201

[16] Walley AY, Cheng DM, Pierce CE, Chen C, Filippell T, Samet JH, Alford DP. methadone dose, take home status, and hospital admission among methadone maintenance patients. J Addict Med. 2012;6(3):186–90. 22694929 10.1097/ADM.0b013e3182584772 PMC3416958

[17] Frank D, Mateu-Gelabert P, Perlman DC, Walters SM, Curran L, Honoria G. “It’s like ‘liquid handcuffs”: the effects of take-home dosing policies on methadone maintenance treatment (MMT) patients’ lives. Harm Reduct J. 2021;18:88. 34391436 10.1186/s12954-021-00535-y PMC8364307

[18] Russell DM. To a US methadone recipient, visiting Australia was shocking. Filter 2022; December 20. [online]: https://filtermag.org/methadone-clinic-australia-pharmacy/ Accessed 12 Feb 2023.

[19] Russell DM. Naturally Non-compliant: mandatory counseling in methadone clinics. (Unpublished doctoral dissertation). Arizona State University. Tempe, Arizona. 2023.

[20] Marshall K, Maina G, Sherstobitoff J. Plausibility of patient-centered care in high-intensity methadone treatment: reflections of providers and patients. Addict Sci Clin Pract. 2021;16:42. 34187549 10.1186/s13722-021-00251-9 PMC8244190

[21] Bowser D, Bohler R, Davis MT, Hodgkin D, Frank RG, Horgan CM. New methadone treatment regulations should be complemented by payment and financing reform. Health Affairs Forefront. 2023. 10.1377/forefront.20230531.974690.

[22] Kessler RC, Berglund P, Demler O, Jin R, Merikangas KR, Walters EE. Lifetime prevalence and age-of-onset distributions of DSM-IV disorders in the national comorbidity survey replication. Arch Gen Psychiatry. 2005;62:593–602. 15939837 10.1001/archpsyc.62.6.593

[23] Villagómez RE, Meyer TJ, Lin MM, Brown LS. Post-traumatic stress disorder among inner city methadone maintenance patients. J Subst Abuse Treat. 1995;12(4):253–7. 8830152 10.1016/0740-5472(95)00025-z

[24] Madden EF. Intervention stigma: how medication-assisted treatment marginalizes patients and providers. Soc Sci Med. 2019;232:324–31. 10.1016/j.socscimed.2019.05.027. 31125801 10.1016/j.socscimed.2019.05.027

[25] National Institutes of Health. 1997. The NIH Consensus Development Program: Effective Medical Treatment of Opiate Addiction [Consensus Development Conference Statement]. https://consensus.nih.gov/1997/1998treatopiateaddiction108html.htm

[26] Joudrey PJ, Chadi N, Roy P, Morford K, Bach P, Kimmel S, Wang EA, Calcaterra SL. Pharmacy-based methadone dispensing and drive time to methadone treatment in five states within the United States: a cross-sectional study. Drug Alco Depend. 2020;211: 107968. 10.1016/j.drugalcdep.2020.107968 PMC7529685 32268248

[27] D’Aunno T, Pollack H. Changes in methadone treatment practices: results from a national panel study, 1988–2000. JAMA. 2002;88(7):850–6. 10.1001/jama.288.7.850 12186602

[28] United States Substance Abuse and Mental Health Services Administration (SAMHSA). FAQs: Provision of methadone and buprenorphine for the treatment of Opioid Use Disorder in the COVID-19 emergency, April 21, 2020. https://tinyurl.com/sxbcnh3

[29] Health and Human Services, Substance Abuse and Mental Health Services Administration. Code of Federal Regulations 42 Part 8: Medications for the Treatment of Opioid Use Disorder. [https://www.federalregister.gov/documents/2024/02/02/2024-01693/medications-for-the-treatment-of-opioid-use-disorder} Accessed 23 April 2024.

[30] Treitler PC, Bowdewn CF, Lloyd J, Enich M, Nyaku AN, Crystal S. Perspectives of opioid use disorder treatment providers during COVID-19: adapting to flexibilities and sustaining reforms. J Sub Ab Treat. 2022;132:108514. 10.1016/j.jsat.2021.108514 PMC8630075 34098210

[31] Krawczyk N. Lessons learned from COVID-19 and regulatory change in the wake of necessity. Report to the National Academies of Science, 2022. https://www.nationalacademies.org/documents/embed/link/LF2255DA3DD1C41C0A42D3BEF0989ACAECE3053A6A9B/file/D3E063A30D9D988E5EB95D66B10AABA47900519FC319

[32] Meyerson BE, Bentele KG, Brady BR, Stavros N, Russell DM, Mahoney A, Garnett I, Jackson S, Garcia RC, Coles H, Granillo B, Carter GA. Insufficient impact: Limited implementation of federal regulatory changes to methadone and buprenorphine access in Arizona during COVID. Am J Prev Med Focus. 2023. 10.1016/j.focus.2023.100177. 10.1016/j.focus.2023.100177 PMC10835120 38312524

[33] Brothers S, Viera A, Heimer R. Changes in methadone program practices and fatal methadone overdose rates in connecticut during COVID-19. J Sub Abuse Treat. 2021;131: 108449. 10.1016/j.jsat.2021.108449 PMC9758251 34098303

[34] Meyerson BE, Bentele KG, Russell DM, Brady BR, Downer M, Garcia RC, Garnett I, Lutz R, Mahoney A, Samorano S, Arredondo C, Andres HJ, Coles H, Granillo B. Nothing really changed: Arizona patient experience of methadone and buprenorphine access during COVID. PLoS ONE. 2022;17(10): e0274094. 36282806 10.1371/journal.pone.0274094 PMC9595554

[35] Jones CM, Shoff C, Hodges K, et al. Receipt of telehealth services, receipt and retention of medications for opioid use disorder, and medically treated overdose among medicare beneficiaries before and during the COVID-19 pandemic. JAMA Psychiat. 2022;79(10):981–92. 10.1001/jamapsychiatry.2022.2284 PMC9434479 36044198

[36] Mandavia AD, Campbell A, Henry BF, et al. Support for COVID-19-related substance use services policy changes: a New York state-wide survey. J Behav Health Serv Res. 2022;49:262–81. 10.1007/s11414-021-09784-y. 35112221 10.1007/s11414-021-09784-y PMC8810146

[37] Fuller BE, Rieckmann T, Nunes EV, Miller M, Arfken C, Edmundson E, McCarty D. Organizational readiness for change and opinions toward treatment innovations. J Sub Abuse Treat. 2007;33:183–92. 10.1016/j.jsat.2006.12.026. 10.1016/j.jsat.2006.12.026 PMC2031859 17434708

[38] Frimpong JA, Guerrero EG, Kong Y, Khachikian T, Wang S, D’Aunno T, Howard DL. Predicting and responding to change: perceived environmental uncertainty among substance use disorder treatment programs. J Sub Use Addic Treat. 2023;145: 208947. 10.1016/j.josat.2022.208947. 10.1016/j.josat.2022.208947 36880916

[39] Pasman E, Lee G, Kollin R, Rodriguez B, Agius E, Madden EF, Resko SM. Attitudes toward medication for opioid use disorder among substance use treatment providers. Subs Misuse. 2022. 10.1080/10826084.2022.211.5853. 10.1080/10826084.2022.2115853 36041008

[40] Aletraris L, Edmond MB, Paino M, Fields D, Roman PM. Counselor training and attitudes toward pharmacotherapies for opioid use disorder. Substance Abuse. 2016;37(1):47–53. 10.1080/08897077.2015.1062457. 26168816 10.1080/08897077.2015.1062457 PMC4879956

[41] Broman MJ, Pasman E, Brown S, Lister JJ, Agius E, Resko SM. Social support is associated with reduced stigma and shame in a sample of rural and small urban adults in methadone treatment. Addict Res Theor. 2022. 10.1080/16066359.2022.2101640.

[42] Cioe K, Biondi BE, Easly R, Simard A, Zheng X, Springer SA. A systematic review of patients’ and providers’ perspectives of medications for treatment of opioid use disorder. J Sub Abuse Treat. 2020;119: 108146. 10.1016/j.jsat.2020.108146 PMC7609980 33138929

[43] Jenkins SR, Mitchell JL, Baird S, Whitfield SR, Meyer HL. The counselor’s trauma as counseling motivation: vulnerability or stress inoculation. J Interpers Violence. 2010;26:2392–412. 20956440 10.1177/0886260510383020

[44] Cosden M, Sanford A, Korch LM, Lepore CE. Vicarious trauma and vicarious posttraumatic growth among substance abuse treatment providers. Sub Abuse. 2016;37(4):619–24. 10.1080/08897077.2016.1181695 27163485

[45] Middleton JS, Potter CC. Relationship between vicarious traumatization and turnover among child welfare professionals. J Public Child Welfare. 2015;9(2):195–216.

[46] McCann IL, Pearlman LA. Vicarious traumatization: a framework for understanding the psychological effects of working with victims. J Traumatic Stress. 1990;3(1):131–49.

[47] Bride BE, Kintzle S. Secondary traumatic stress, job satisfaction, and occupational commitment in substance abuse counselors. Traumatology. 2011;17:22–8.

[48] Peavy KM, Darnton J, Grekin P, Russo M, Banta Green CJ, Merrill JO, Fotinos C, Woolworth S, Soth S, Tsui JI. Rapid implementation of service delivery changes to mitigate COVID-19 and maintain access to methadone among persons with and at high risk for HIV in an opioid treatment program. AIDS Behav. 2020;24:2469–72. 32347404 10.1007/s10461-020-02887-1 PMC7186943

[49] Hein DA, Nunes E, Levin FR, Fraser D. Posttraumatic stress disorder and short-term outcome in early methadone treatment. J Sub Abuse Treat. 2000;19:31–7. 10.1016/s0740-5472(99)00088-4 10867298

[50] Ecker AH, Hundt N. Posttraumatic stress disorder in opioid agonist therapy: a review. Psychol Trauma. 2018;10(6):636. 28758767 10.1037/tra0000312

[51] Linde-Krieger LB, Meyerson BE, Huff AJ, Carter GA. An exploratory study of trauma, vicarious trauma, and lived substance use disorder among opioid treatment program staff: implications for staff functioning. (in review)

[52] Agley JD, Meyerson BE, Eldridge LA, Jun M, Vadiei N, Crosby RA, Bentele KG, Kennedy A, Anderson K. Exploration of pharmacist comfort with harm reduction behaviors: cross-sectional latent class analysis. J Am Pharm Assoc. 2022;62(2):432–40. 10.1016/j.japh.2021.10.015. 10.1016/j.japh.2021.10.015 34742654

[53] Meyerson BE, Agley JD, Jayawardene W, Eldridge LA, Arora P, Smith C, Vadiei N, Kennedy A, Moehling T, PharmNet Research Team. Feasibility of a pharmacy-based harm reduction intervention to reduce opioid overdose, HIV and hepatitis C—Indiana 2019. Res Soc Admin Pharm. 2020;16(5):699–709. 10.1016/j.sapharm.2019.08.026. 10.1016/j.sapharm.2019.08.026 31611071

[54] Jayawardene W, Carter GA, Agley JD, Meyerson BE, Garcia J, Miller W. HIV pre-exposure prophylaxis uptake by advanced practice nurses: Interplay of agency, community and attitudinal factors. J Adv Nursing. 2019;75(11):2559–69. 10.1111/jan.14019. 10.1111/jan.14019 30950528

[55] Brady B, Meyerson BE, Bentele KG. Flying blind: survey research among methadone and buprenorphine providers in Arizona. Survey methods: insights from the field 2023; https://surveyinsights.org/?p=17985

[56] Baker CN, Brown SM, Wilcox PD, Overstreet S, Arora P. Development and psychometric evaluation of the attitudes related to trauma-informed care (ARTIC) Scale. Sch Ment Heal. 2016;8(1):61–76.

[57] Yang LH, Grivel MM, Anderson B, Bailey GL, Opler M, Wong LY, Stein MD. A new brief opioid stigma scale to assess perceived public attitudes and internalized stigma: evidence for construct validity. J Subst Abuse Treat. 2019;99:44–51. 10.1016/j.jsat.2019.01.005. 30797393 10.1016/j.jsat.2019.01.005 PMC6716158

[58] Weathers FW, Blake DD, Schnurr PP, Kaloupek DG, Marx BP, Keane TM. The life events checklist for DSM-5 (LEC-5). Instrument available from the National Center for PTSD at www.ptsd.va.gov. 2013.

[59] Felitti VJ, Anda RF, Nordenberg D, Williamson DF, Spitz AM, Edwards V, Koss MP, Marks JS. Relationship of childhood abuse and household dysfunction to many of the leading causes of death in adults. The adverse childhood experiences (ACE) Study. Am J Prev Med. 1998;14(4):245–58. 10.1016/s0749-3797(98)00017-8. 9635069 10.1016/s0749-3797(98)00017-8

[60] Blevins CA, Weathers FW, Davis MT, Witte TK, Domino JL. The posttraumatic stress disorder checklist for DSM-5 (PCL-5): development and initial psychometric evaluation. J Trauma Stress. 2015;28(6):489–98. 10.1002/jts.22059. 26606250 10.1002/jts.22059

[61] Briere J. Psychometric review of the trauma symptom checklist-40, in B.H. Stamm (Ed). Measurement of stress, trauma, and adaptation. Lutherville, MD: Sidran Press, 1996. http://s1097954.instanturl.net/trauma-symptom-checklist-40-tsi-40/

[62] Benuto L, Singer J, Cummings C, Ahrendt A. The vicarious trauma scale: confirmatory factor analysis and psychometric properties with a sample of victim advocates. Health Soc Care Commun. 2018;26(4):564–71. 10.1111/hsc.12554 29488272

[63] Wilberforce M, Sköldunger A, Edvardsson D. A Rasch analysis of the person-centered climate questionnaire—staff version. BMC Health Serv Res. 2019;19:996. 10.1186/s12913-019-4803-9. 31878914 10.1186/s12913-019-4803-9 PMC6933628

[64] Cai L, Ahlström G, Tang P, Ma K, Edvardsson D, Behm L, Fu H, Zhang J, Yang J. Psychometric evaluation of the Chinese version of the person-centred climate questionnaire—staff version (PCQ-S). BMJ Open. 2017;7(8):017250. 10.1136/bmjopen-2017-017250 PMC5724089 28851797

[65] Yoon JY, Roberts T, Grau B, Edvardsson D. Person-centered climate questionnaire-patient in English: a psychometric evaluation study in long-term care settings arch of. Gerontol Geriatrics. 2015;61(1):81–7. 10.1016/j.archger.2015.03.010 25865746

[66] National Harm Reduction Coalition. Foundational Principles Central to Harm Reduction. https://harmreduction.org/about-us/principles-of-harm-reduction/ Accessed 23 April 2024.

[67] Briggs EC, Amaya-Jackson L, Putnam KT, Putnam FW. All adverse childhood experiences are not equal: the contribution of synergy to adverse childhood experience scores. Am Psychol. 2021;76(2):243. 33734792 10.1037/amp0000768

[68] Doukas N, Cullen J. Recovered addicts working in the addiction field: pitfalls to substance abuse relapse. Drugs Educ Prev Policy. 2010;17:216–31.

[69] Humphreys K, Noke J, Moos RH. Recovering substance abuse staff members’ beliefs about addiction. J Sub Abuse Treat. 1996;13(1):75–8. 10.1016/0740-5472(95)02019-5 8699546

[70] Caplehorn JR, Irwig L, Saunders JB. Attitudes and beliefs of staff working in methadone maintenance clinics. Subst Use Misuse. 1996;31(4):437–52. 10.3109/10826089609045820. 8851811 10.3109/10826089609045820

[71] University of Michigan Behavioral Health Workforce Research Center. National analysis of peer support providers: Practice settings, requirements, roles and reimbursement. Ann Arbor, MI: UMSPH, 2019. [online]: https://www.behavioralhealthworkforce.org/wp-content/uploads/2019/10/BHWRC-Peer-Workforce-Full-Report.pdf Accessed 25 May 2023.

[72] Wang CL, Ahmed PK. Organizational learning: a critical review. Learning Org. 2003;10(1):8–17.

[73] Damschroder LJ, Hagedorn HJ. A guiding framework and approach for implementation research in substance use disorders treatment. Psych of Addic Behav. 2011;25(2):194–205. 10.1037/a0022284 21443291

[74] Zoom discussion about findings with OTP leadership of sample clinics. July 5, 2023.

